# Linking entities through an ontology using word embeddings and syntactic re-ranking

**DOI:** 10.1186/s12859-019-2678-8

**Published:** 2019-03-27

**Authors:** İlknur Karadeniz, Arzucan Özgür

**Affiliations:** Department of Computer Engineering, Boğaziçi University, İstanbul, 34342 Turkey

**Keywords:** Text mining, Natural language processing, Named entity normalization, Entity linking, Entity categorization, Bacteria biotopes, Adverse drug reactions, Word embeddings

## Abstract

**Background:**

Although there is an enormous number of textual resources in the biomedical domain, currently, manually curated resources cover only a small part of the existing knowledge. The vast majority of these information is in unstructured form which contain nonstandard naming conventions. The task of named entity recognition, which is the identification of entity names from text, is not adequate without a standardization step. Linking each identified entity mention in text to an ontology/dictionary concept is an essential task to make sense of the identified entities. This paper presents an unsupervised approach for the linking of named entities to concepts in an ontology/dictionary. We propose an approach for the normalization of biomedical entities through an ontology/dictionary by using word embeddings to represent semantic spaces, and a syntactic parser to give higher weight to the most informative word in the named entity mentions.

**Results:**

We applied the proposed method to two different normalization tasks: the normalization of bacteria biotope entities through the Onto-Biotope ontology and the normalization of adverse drug reaction entities through the Medical Dictionary for Regulatory Activities (MedDRA). The proposed method achieved a precision score of 65.9%, which is 2.9 percentage points above the state-of-the-art result on the BioNLP Shared Task 2016 Bacteria Biotope test data and a macro-averaged precision score of 68.7% on the Text Analysis Conference 2017 Adverse Drug Reaction test data.

**Conclusions:**

The core contribution of this paper is a syntax-based way of combining the individual word vectors to form vectors for the named entity mentions and ontology concepts, which can then be used to measure the similarity between them. The proposed approach is unsupervised and does not require labeled data, making it easily applicable to different domains.

## Background

Currently, the vast majority of the biomedical resources are in unstructured form which originate from an assortment of different resources that incorporate nonstandard naming conventions, which makes the required information difficult to use and understand [10]. Ontologies help researchers to overcome these kinds of difficulties and help researchers facilitate the vast amounts of biomedical knowledge available [41]. An ontology can provide a unique identifier for describing information for each entity, which solves the heterogeneity problem and provides standardized and homogeneous data [39].

Linking named entities in text through an ontology is an essential process to make sense of the identified named entities [11]. When an ontology/dictionary containing a set of entities *E* and a text containing a set of entity mentions *M* are given, entity linking is the task of mapping each named entity mention *m* in the given text to its corresponding entity *e* in the given ontology/dictionary, where *m*∈*M* and *e*∈*E* [40]. This task is also called entity normalization, entity grounding, or entity categorization, which are used interchangeably throughout this paper.

**Fig. 1:**
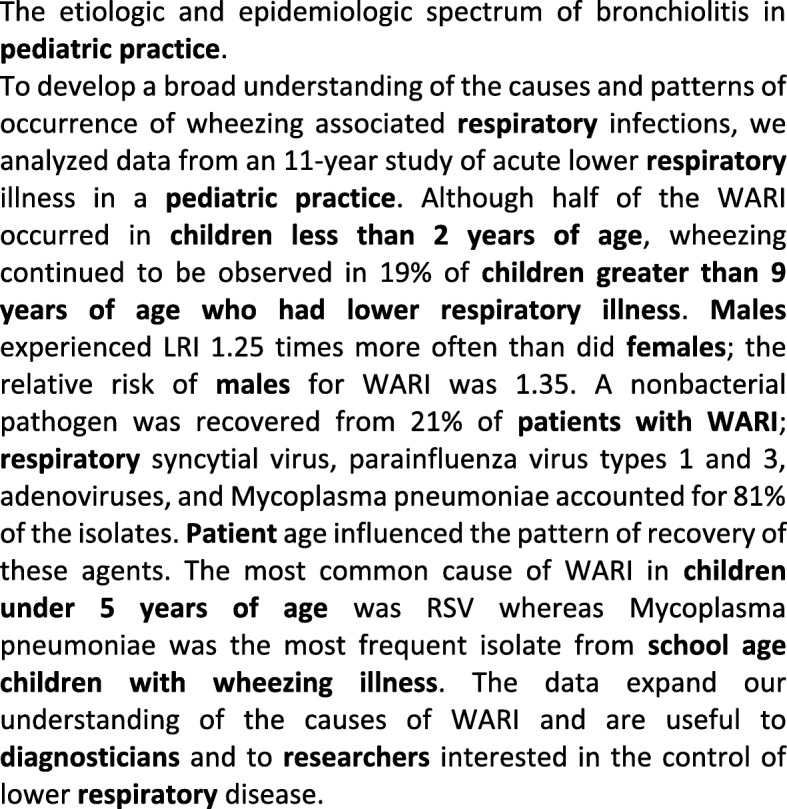
Sample text. Sample abstract of [21] with habitat entity mentions annotated

**Fig. 2:**
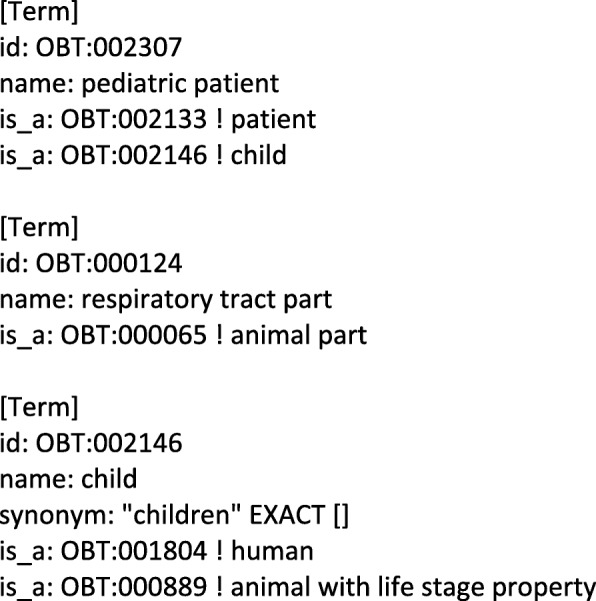
Sample ontology. A sample portion from the Onto-Biotope ontology

Figure 1 demonstrates a sample text with annotated bacteria habitat (biotope) mentions, which are represented in bold and Fig. 2 demonstrates a sample portion from Onto-Biotope, which is an ontology for bacteria habitats. Given a sample text with annotated habitat mentions, the aim of habitat entity normalization is to link the mentions through the Onto-Biotope Ontology. For instance, *“pediatric”*, *“respiratory”*, and *“children less than 2 years of age”* are habitat entity mentions. The concept that is associated with the *“pediatric”* habitat mention in the Onto-Biotope ontology is *“pediatric patient”*, the one associated with the *“respiratory”* habitat mention is *“respiratory tract part”*, and for *“children less than 2 years of age”* it is *“pediatric patient”*.

The association between the entity mention *“pediatric”* and the ontology concept term name *“pediatric patient”* can be relatively more easily detected due to the lexical similarity between them. Similarly, the habitat mention *“respiratory”* and the ontology concept *“respiratory tract part”* also share a common word, making them lexically similar. However, lexical similarity may not always exist between entity mentions and concept term names or concept synonyms. For example, there is no lexical similarity between the habitat mention *“children less than 2 years of age”* and ontology concept term name *“pediatric patient”*, which calls for the utilization of semantic similarity.

Entity normalization can also be performed through a dictionary. For instance, the sample sentence *“In Study 3, 67% of patients treated with ADCETRIS experienced any grade of neuropathy.”* states a relation between the drug mention *“ADCETRIS”* and adverse drug reaction mention *“neuropathy”*. The adverse drug reaction mention *“neuropathy”* can be normalized to the *“peripheral neuropathy”* term in the Medical Dictionary for Regulatory Activities (MedDRA) [7].

Even if the named entities are given, linking the identified named entities to a unique concept identifier in an ontology/dictionary is not a trivial task in the biomedical domain. There are many challenges in the task of named entity linking through an ontology or a dictionary, two of which are the variety and ambiguity problems of the named entities [4]. A named entity may appear in different surface forms in a given text, which is called the variety problem. Furthermore, two named entities with the same surface form may have different semantic meanings, which is called the ambiguity problem. Linking of named entities for the biomedical domain has another big challenge besides these two common problems in the general natural language processing domain. In the biomedical domain, the training data is relatively smaller and the number of the ontology/dictionary categories that should be considered is larger compared to many other domains in natural language processing [6]. This poses a challenge for the standard supervised classification algorithms. For example, there are 2,221 semantic categories in the Onto-Biotope ontology, while the available training set contains only 747 entity mentions, and 16,295 words. For adverse drug reaction normalization, this situation is worse since there are 22,499 MedDRA dictionary terms.

In this paper, for the ontology based normalization of the named entity mentions in text, we propose an unsupervised approach, which utilizes both semantic and syntactic information. The proposed approach uses word embeddings learned from large unlabeled text to capture semantic information and syntactic parsing information to re-rank the candidate ontology/dictionary concept terms. The proposed approach is tested on two different data sets, which are the BioNLP Shared Task 2016 Bacteria Biotopes (BB3) categorization sub-task data to normalize habitat entities through the Onto-Biotope ontology and the Text Analysis Conference 2017 Adverse Drug Reaction data to normalize adverse drug reaction mentions through the MedDRA dictionary. On both data sets, the proposed normalization method with syntactic re-ranking achieved better performance than the normalization method without syntactic re-ranking. Furthermore, we obtained the new state-of-the-art results with 2.9 percentage points above the previous best result for the Bacteria Biotopes (BB3) categorization sub-task.

### Related work

Several approaches have been proposed for biomedical entity normalization for different types of biomedical entities including genes/proteins [20, 32, 36, 46], bacteria biotopes [6, 13, 23, 37, 43], and diseases [14, 28]. Early systems tried to link the entity mentions to the knowledge base entities by utilizing dictionary look-up and string matching algorithms [16, 36]. Some studies [14, 23] used hand-written rules to measure the morphological similarity between entity mentions and ontology/dictionary entities, while others [17] automatically learned patterns of variations of the entities. Machine-learning based approaches, which learn the similarities between biomedical entity mentions and ontology concept names from labeled training data have also been proposed and applied as a solution to the normalization task of various biomedical entities such as diseases [28].

Most previous studies focused on utilizing morphological information for named entity normalization. However, morphological similarity alone is not adequate to normalize biomedical entities, which generally have forms different from the concept terms that they should be tagged with [6]. Word embedding models, which learn distributed representations of words from large unlabeled corpora, are promising approaches for capturing semantic information [34]. They have been successfully used in several recent Natural Language Processing (NLP) tasks including the biomedical domain [3, 8, 35, 42]. Recently, word embeddings have also been used for the task of biomedical named entity normalization. Li et al. [30] proposed a convolutional neural network (CNN) architecture leveraging semantic and morphological information, which handles the biomedical entity normalization task as a ranking problem. In the proposed method, firstly candidates are generated using hand-crafted rules, and then they are ranked according to semantic and morphological information, which are represented by a CNN-based model. Experiments on two benchmark datasets (the ShARe/CLEF eHealth dataset and the NCBI disease dataset) showed that semantic information is beneficial for the biomedical entity normalization task as well as morphological information. However, the requirement of hand-crafted rules and labeled data makes the adaptation of this method to different domains harder and time-consuming. Cho et al. [9] proposed a semi-supervised approach that facilitates word embeddings to represent semantic spaces for normalizing biomedical entities such as disease names and plant names and obtained promising performance. This method requires a domain specific corpus and dictionary. Therefore, the adaptation of it to other domains is not easy, if there are no such resources available.

A number of community-wide challenges including the BioCreative Challenges [1, 2, 22, 29, 47] and BioNLP Shared Tasks [13, 24, 25, 37], which have been conducted to assist the progress of research in biomedical text mining, also addressed the task of biomedical entity normalization. The Bacteria Biotope task, whose ultimate aim is information extraction regarding bacteria and their habitats, was first addressed in the BioNLP Shared Task 2011 [5, 25], and has been conducted in 2013 [6, 37] and 2016 again since then. We evaluated our proposed approach on the BB-cat subtask of the 2016 edition of the Bacteria Biotope task, which addressed the normalization of habitat entity mentions in PubMed abstracts using the OntoBiotope ontology [13]. In the official task, the teams TagIt [12] and LIMSI [18] proposed rule-based methods, while BOUN [43] proposed a similarity-based method that utilizes both approximate string matching and cosine similarity of word-vectors weighted with Term Frequency-Inverse Document Frequency (TF-IDF). According to the official results, the best precision (62%) for habitat mention normalization was obtained by the BOUN system.

The bacteria habitat mention normalization problem continued to attract the attention of the researchers after the shared task. CONTES is a recently proposed semi-supervised method for linking habitat entity mentions through the Onto-Biotope ontology [15]. The system is based on word embeddings that are induced from PubMed by utilizing the Word2Vec tool. The cosine similarities between term vector representations and concept vector representations are calculated to find the most similar ontology concept to the given entity mention. They applied the proposed normalization method to the test dataset of the Bacteria Biotope 2016 Task 3 (BB-cat), and obtained comparable results to that of the state-of-the-art for the task of Bacteria Biotopes categorization. CONTES contains a transformation step to make comparable the term vectors and the entity vectors which are represented in different dimensions. The need for the transformation step makes the method semi-supervised, since it requires labeled data for training the prediction model. Recently, Mehryary et al. [33] used TF-IDF weighted vector space representation for the named entity categorization of bacteria biotopes. Each ontology concept name and each entity mention is represented with a TF-IDF weighted vector considering each concept name in the ontology as a separate document and calculating IDF weights based on these names. The ontology concept with the highest cosine similarity is assigned to a given entity mention. Although they achieved state-of-the-art results in the normalization task, the TF-IDF based scheme has limitations in capturing the semantic relations between the ontology concepts and entity mentions, since it is primarily based on the surface forms of the words.

Besides the Bacteria Biotopes normalization task, we also evaluate our approach on the task of normalizing Adverse Drug Reaction (ADR) mentions in drug labels to the MedDRA terms. We use the recently provided data set from the Text Analysis Conference (TAC) 2017. Different types of data sources such as electronic health records [19], scientific publications, and social media data [38] and different types of lexicons such as the Unified Medical Language System (UMLS) [31] and the side effect resource (SIDER) [44] have been used to extract ADRs from text. Many of these studies proposed a lexicon-based matching approach for ADRs recognition. Although a number of studies have been conducted to automatically identify ADRs in text and map them through a dictionary using NLP techniques, as far as we know the normalization of the ADRs through a dictionary has not been studied as a separate task without named entity recognition.

## Methods

**Fig. 3:**
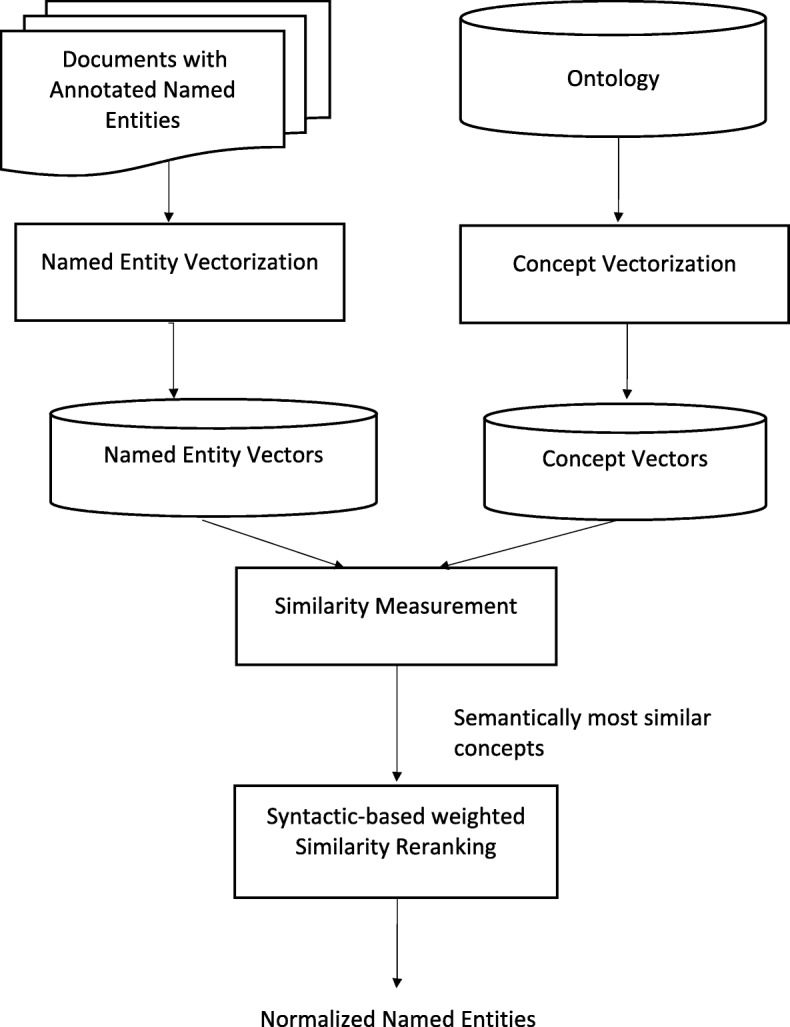
System Workflow. Workflow of the Named Entity Normalization System

We developed a semantic similarity based unsupervised method for entity linking through an ontology/dictionary, the workflow of which is displayed in Fig. 3. Given a set of documents with annotated named entities and a corresponding ontology, the normalization task is done in two steps. In the first step, the semantically most similar ontology concepts are generated as candidates, and in the second step, the candidates are re-ranked according to the syntactic-based weighted semantic similarities. The details of our approach are described in the following subsections.

### Data sets

#### Bacteria biotope entity normalization

In this study, we used the official data set that is provided by the BioNLP Shared Task 2016 organizers for the Bacteria Biotope categorization subtask. Since our proposed approach is unsupervised and does not require any training data, the training and development sets are used for error analysis during the development of the system, and the separate test set is used for evaluating the performance of the proposed system. The data set provided by the shared task organizers was created by collecting titles and abstracts from PubMed, which contain general information about bacteria and habitats. The data set, consisting of 71 training, 36 development, and 54 test documents, was manually annotated by the bioinformaticians of the Bibliome team of MIG Laboratory at the Institut National de Recherche Agronomique (INRA) [13].

#### Adverse drug reaction normalization

For Adverse Drug Reaction Normalization, we used the official data set that is provided by the Text Analysis Conference (TAC) 2017 organizers. The test set is used for evaluating the performance of the proposed system. The data set contains general information about drug labels consisting of 101 training and 99 test documents, which were manually annotated by the organizers.

### Preprocessing

In the preprocessing step, the annotated named entities and the ontology concept names with their synonyms are tokenized, and the stop words are removed from the named entity mentions and the ontology concept names. Furthermore, all non-ASCII characters are stripped from both the named entities and the ontology concept names.

### Word representations

Our proposed approach is mainly based on the assumption that semantically similar words have similar vector spaces. Based on this assumption, if the semantic similarity of named entity mentions and ontology concept terms can be computed, the most similar concept in the ontology can be assigned as the normalized concept to the named entity mention.

To compute the semantic similarity, each word is represented in the vector space as a real-valued vector using a pre-trained word embedding model that is publicly available [8]. The model has been trained leveraging word vectors that were induced from PubMed by the Word2Vec tool [34]. The trained model is applied to each word to obtain the corresponding word vector. We used the model variant with window size of 30, since it has been shown to obtain higher performance in the biomedical concept similarity and relatedness tasks in the previous study by Chiu et al. [8].

### Identifying the semantically similar ontology concepts

**Fig. 4:**
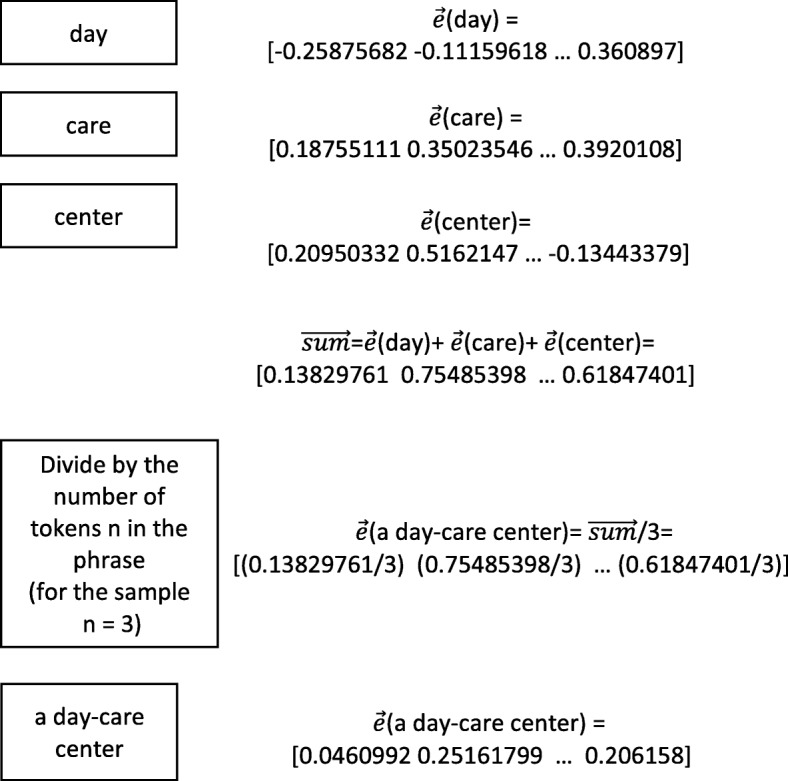
Sample multi-word expression. Computation of the corresponding real-value vector for a sample multi-word expression *“a day-care center”*, where $\vec {e}$(t) is the word embedding vector for token t

The vectors of the ontology concept terms and the reference named entities (i.e., the named entity mentions in text) are computed in the same way as described below. For each word in the named entities and ontology concept terms, the vector representations are obtained by the pre-trained model as explained in the previous subsection. For the multi-word named entities and ontology concepts, the vector representations are computed by averaging the vectors of their composing words. Figure 4 presents the computation of the vector representation for a sample multi-word named entity *“a day-care center”* and shows how the averaging is done. In the preprocessing step, the stop-word *“a”* and the hyphen character are removed. The tokens *“day”*, *“care”*, and *“center”* are considered and used for averaging to compute the vector representation of the multi-word named entity. Each token is represented with a real-valued vector using the pre-trained word embedding model that is explained in the previous subsection. The real-valued vectors of the tokens comprising the multi-word entity mention are summed to create a real-valued vector, which is called $\protect \vec {sum}$. At the end, $\protect \vec {sum}$ is divided by the number of tokens other than the stop-words, which is 3 for the example entity mention, to obtain a normalized real-valued vector for the multi-word named entity.

For each reference entity and for each ontology concept term, a cosine similarity score is calculated to get the semantic similarity between the related entity and the ontology concept term. Since the vectors of ontology concept terms and reference named entities are computed in the same way, unlike the CONTES system, there is no need for a transformation step for the vectors in order to compute the similarity between them. For each reference entity, ontology terms are ranked according to the semantic similarity scores, the top *k* of which are the candidates for syntactic weighting based re-ranking.

We also investigated using word mover’s distance (WMD), instead of cosine similarity. WMD is a distance metric which represents text documents as a weighted point cloud of embedded words and computes the distance between documents as the minimum cumulative distance that words from a document need to travel to another [27]. It is based on the idea that documents without common words may convey similar meanings and bag-of-words (BOW) is not enough to detect this kind of similarity.

### Syntactic re-ranking

Our system without syntactic analysis is not adequate alone to normalize entity mentions like *“children attending a day-care center”*. Table 1 (Before re-ranking part) shows the output of our system without syntactic re-ranking for the sample entity mention *“children attending a day-care center”*. The semantically most similar concepts to the mention are found as *“OBT:001423 medical center”*, *“OBT:001801 clinic”*, and *“OBT:000259 research and study center”*, which are false positives. The correct concept is *“OBT:002146 child”*, which is very similar to the head word *“children”* of the mention *“children attending a day-care center”*. As this example shows, if the system can identify the most informative word in the reference entity mention, the correct concept can be assigned to it (see Table 1 (After re-ranking part)).

**Table 1:** Semantically most similar concepts to the entity mention *“children attending a day-care center”* with/without re-ranking

Rank	Concept	Similarity score
Before Re-ranking		
1	OBT:001423 medical center	0.8297
2	OBT:001801 clinic	0.7917
28	OBT:002146 child	0.6979
After Re-ranking		
1	OBT:002146 child	0.7484
3	OBT:001801 clinic	0.6519
24	OBT:001423 medical center	0.5460

We proposed a re-ranking module based on syntactic parsing to identify the correct concept from among the top *k* candidates returned by the word-embedding based similarity ranking. The re-ranking module makes use of the Stanford Parser (version 3.8.0) [26] to detect the most informative word in the reference entity mention. It computes the semantic similarity between the most informative words of the reference mention and the candidate ontology concept, and re-ranks the top *k* semantically most similar concepts.

**Fig. 5:**
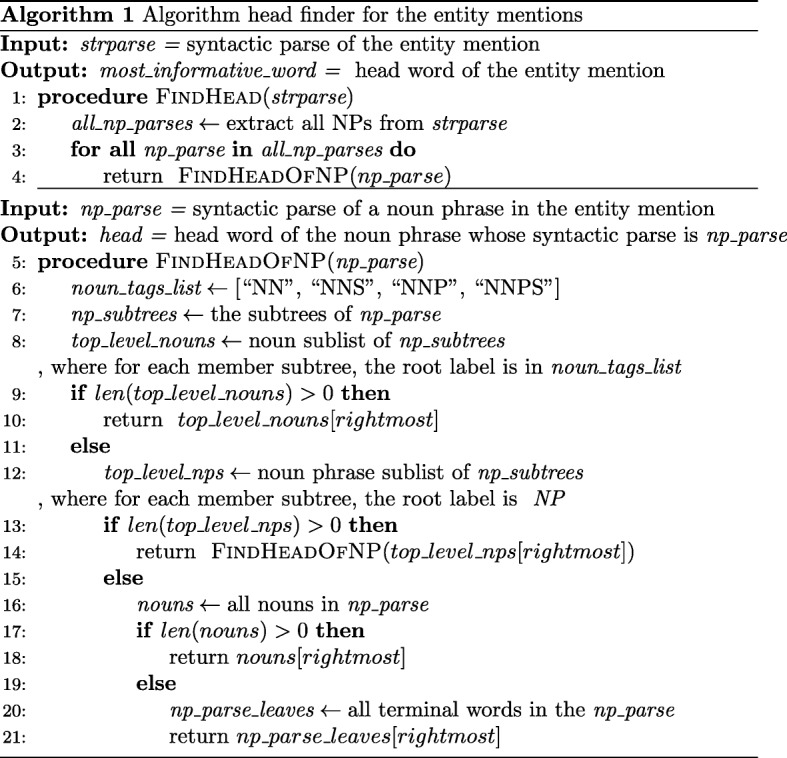
Pseudo-code. Algorithm for finding the most informative word in an entity mention whose syntactic parse is given as input. NP: Noun Phrase; NN: Noun singular; NNS: Noun plural ; NNP: Proper noun singular; NNPS: Proper Noun plural

**Fig. 6:**
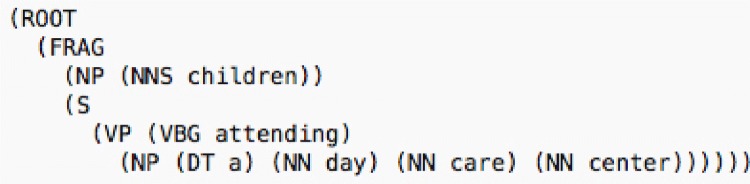
Sample syntactic parse. Syntactic parse of the Stanford Parser for the sample named entity mention *“children attending a day-care center”*

**Fig. 7:**
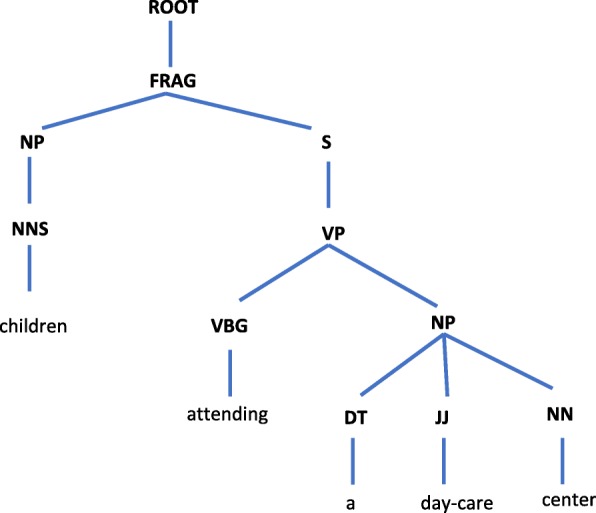
Tree view of the sample parse. Tree view of the syntactic parse of the sample named entity mention *“children attending a day-care center”*

The intuition behind our re-ranking approach is that the entity mentions are noun phrases and the heads of the noun phrases are the most informative words in the mentions. To obtain the corresponding head words, the part-of-speech tags and syntactic parses of the entity mentions are required. We used the Stanford Parser by providing the entity mentions as input and obtaining the syntactic parses composed of their constituent phrases as output. Next, the syntactic parses are processed to find the most informative words in the mentions by utilizing the algorithm whose pseudo-code is given in Fig. 5. According to this algorithm, the top level rightmost *“noun”* is searched in the tree structured syntactic parse and assigned as the head of the mention phrase. For example, for the sample mention *“children attending a day-care center”*, the Stanford Parser generates the syntactic parse, which is shown in Figs. 6 and 7. Figure 6 demonstrates the syntactic parse with its constituent phrases and Fig. 7 shows the tree view. The head of the sample mention is found as *“children”* and the head of the concept name *“OBT:001423 medical center”* is found as *“center”* by leveraging the algorithm.

After the detection of the head words of the phrases as *“children”* for the *“children attending a day-care center”* entity mention and *“center”* for the *“OBT:001423 medical center”* ontology concept name, the semantic similarities are recomputed based on these new information. The similarity scores of the concepts with unrelated head words (e.g. *“OBT:001423 medical center”*) will be lower and those of concepts with related head words (e.g. *“OBT:002146 child”*) will be higher after the re-ranking phase (see Table 1).

The mathematical formulation of the syntactic weighting based similarity used for re-ranking is shown in Equation (1), where *S*_*RR*_(*m*,*c*) is the final computed similarity between mention *m* and candidate concept *c*, and *S*_*S*_ is the semantic similarity, in which *m*_*head*_ is the head word of the mention *m* and *c*_*head*_ is the head word of the concept *c*, *S*_*S*_(*m*,*c*) is the similarity between mention *m* and concept *c* computed as described in “Identifying the semantically similar ontology concepts” section, and *w* is a weighting parameter which can take values between 0 and 1.

1 $$ {S_{RR} (m, c)} = {(w * S_{S}(m_{head}, c_{head}))} + {((1-w) * S_{S}(m, c))}   $$

## Results

In this section, the results of the proposed systems both with and without re-ranking are presented.

### Evaluation metrics

#### Evaluation for bacteria biotopes

For evaluation of the bacteria biotopes entity normalization predictions, we used the official on-line evaluation service to compute the precision score, which is the official measure used to rank the submissions in the BioNLP Shared Task 2016 Bacteria Biotopes categorization sub-task.

In the BioNLP Shared Task 2016 Bacteria Biotopes categorization sub-task, entities have been given and the participants were required to predict the normalization of the entities. In the official on-line evaluation, for each normalized Habitat entity, Wang similarity *W* [45] is calculated with s = 0.65 to measure the similarity between the reference and the predicted normalization. Wang similarity is the Jaccard index between the two sets of the predicted and the reference concept ancestors with a weighted factor *d*^*s*^, where *d* is the distance between the corresponding concept and the ancestor, and *s* is a parameter between 0 and 1. The submissions are evaluated with their Precision values:

2 $$ {Precision} = {\sum} {S_{p} / {N}}   $$

where *S*_*p*_ is the total Wang similarity *W* for all predictions [13], and *N* is the number of predicted entities.

#### Evaluation for adverse drug reaction

For evaluation of the adverse drug reactions entity normalization predictions, we computed the macro-averaged and micro-averaged scores for precision, recall and f-score measures. True positives (TP), false positives (FP), and false negatives (FN) are calculated by comparing the predicted normalization concept with the reference normalization concept in the gold standard via exact matching.

To compute Micro-average scores, the true positives, false positives, and false negatives of the system are summed up for all drug labels to get the statistics (Eqs. 3 and 4). *N* is the total number of drug labels in the data set.

3 $$ {Micro-average\ Precision} = \frac{\sum\limits_{c=1}^{N}({TP}_{c})}{\sum\limits_{c=1}^{N}({TP}_{c} + {FP}_{c})}   $$

4 $$ {Micro-average\ Recall} = \frac{\sum\limits_{c=1}^{N}({TP}_{c})}{\sum\limits_{c=1}^{N}({TP}_{c} + {FN}_{c})}   $$

On the other hand, the macro-averaged scores are computed as the average of the individual precision and recall values obtained on each drug label (Eqs. 5 and 6).

5 $$ {Macro-average\ Precision} = \frac{\sum\limits_{c=1}^{N}({Precision}_{c})}{(N)}   $$

6 $$ {Macro-average\ Recall} = \frac{\sum\limits_{c=1}^{N}({Recall}_{c})}{(N)}   $$

### Results for bacteria biotopes

Table 2 shows the results of our proposed approach with and without syntactic re-ranking. The results show that the system with the syntactic re-ranking module achieves a higher performance. Recall that the proposed system without re-ranking computes the vector representations for the multi-word entities by averaging the vectors of their composing words. On the other hand, the proposed system with syntactic re-ranking computes the vector representations by giving higher weights to the head words. This means that instead of averaging the vector representations, giving higher weights to the most informative words is a more suitable way for vector representations of multi-word entities.

**Table 2:** Results for the system with and without syntactic re-ranking

System	Train	Dev
Before Re-ranking	0.601	0.629
After Re-ranking	0.648	0.677

Precision values for the training and development data sets are reported. *k* is set to 5 and *w* is set as 0.25 for the re-ranking module

Table 3 presents a comparison of the proposed system, named as BOUNEL (BOUN Named Entity Linker), with the prior work on the task of habitat named entity normalization. We compared our results with the previous systems that are tested on the BioNLP Shared Task 2016 BB cat subtask test set. We computed two different baseline results; the BASELINE-1 assigns the exact match of the term in the ontology. In case of non-existence of an exact match, BASELINE-1 assigns the term to the root concept of the Onto-Biotope ontology hierarchy, which is “bacteria habitat” concept. On the other hand, BASELINE-2 assigns all terms to the “bacteria habitat” concept without searching for an exact match. The results show that our system obtained a score of 65.9% which is higher than both of the baselines BASELINE-1 and BASELINE-2. Our proposed method also obtained higher scores than all other previously proposed methods on the bacteria biotope normalization task, achieving the new state-of-the-art results.

**Table 3:** Comparison with previous systems for the normalization task of bacteria biotopes

System	Precision
BOUNEL(Our system)	0.659
TURKU [33]	0.630
BOUN [43]	0.620
CONTES [15]	0.597
LIMSI [18]	0.438
BASELINE-2	0.322
BASELINE-1	0.225

Precision values for the test data set are reported. *k* is set to 5 and *w* to 0.25 for the proposed system (BOUNEL) based on the results on the training and development sets

### Results for adverse drug reactions

Table 4 presents the results of the proposed system before and after syntactic re-ranking for the task of adverse drug reactions entity normalization on the Text Analysis Conference 2017 Adverse Drug Reaction training and test data sets. We used the same values for the parameters of the re-ranking module as the bacteria biotope normalization task (k=5 and w=0.25). Since there is no prior work on the task of adverse drug reactions entity normalization task on the same data set, we compared our results with the baseline. We computed baseline results by assigning the mention to the exact match of the term in the MedDRA dictionary. As the results on Table 4 demonstrate, the new system with syntactic re-ranking obtained higher precision, recall, and f-measure scores on both the training and test data sets than the system without syntactic re-ranking. Furthermore, the new system with syntactic re-ranking achieved significantly higher recall than the baseline, as a result achieving higher f-measure scores.

**Table 4:** Results of the proposed method with/without re-ranking on the adverse drug reaction normalization task

	Baseline	Before Re-ranking	After Re-ranking
Training set			
Macro-average Precision	0.999	0.737	0.742
Macro-average Recall	0.522	0.732	0.736
Macro-average F-score	0.686	0.735	0.739
Micro-average Precision	0.999	0.728	0.730
Micro-average Recall	0.513	0.723	0.725
Micro-average F-score	0.665	0.726	0.728
Test set			
Macro-average Precision	0.999	0.683	0.687
Macro-average Recall	0.494	0.677	0.681
Macro-average F-score	0.661	0.675	0.684
Micro-average Precision	0.999	0.682	0.686
Micro-average Recall	0.489	0.675	0.680
Micro-average F-score	0.657	0.678	0.684

Precision, recall and f-score values for the training and test sets are reported

## Discussion

### Bacteria biotopes

Table 5 shows the performance of the proposed system without syntactic re-ranking for returning the correct concept from the ontology among the top *k* ranked candidates. For example, when *k*=1, the concept assignment is considered correct, only if the correct concept is ranked first by the system. On the other hand, when *k*=10, the concept assignment is considered correct, if the correct concepts is ranked in the top ten by the system. These results motivated the development of the re-ranking module, since as *k* increases, the precision of the system also increases. The goal of syntactic re-ranking is to re-rank the top *k* retrieved candidate concepts, so that the correct concept moves to the first rank, as in the example shown in Table 1.

**Table 5:** Prediction performance of our system without syntactic re-ranking among the semantically most similar top (k = 1, 5, 10, 20, 25, 50) concepts

k	1	5	10	15	20	25	50
Train	0.614	0.656	0.672	0.711	0.726	0.738	0.831
Dev	0.655	0.683	0.725	0.753	0.789	0.804	0.894

Precision values for the training and development data sets are reported when the reference concept is among the top k

Table 6 demonstrates the results of our proposed approach with syntactic re-ranking, when the top *k* candidates retrieved by the system without re-ranking are provided as input to the re-ranking module. As the results show, for values of *k*=10,*k*=15,*k*=20 and *k*=25, the results are nearly the same on the training and development sets, which means that after a threshold of *k*=5, different values of *k* make no big difference in the results. Therefore, based on the results on the training and development sets, *k* is chosen as *5* empirically.

**Table 6:** Results for the system with syntactic re-ranking for the different number of semantically most similar top (k = 5, 10, 15, 20, 25, 50) concepts

k	5	10	15	20	25	50
Train	0.648	0.634	0.637	0.639	0.640	0.643
Dev	0.677	0.668	0.667	0.667	0.668	0.632

Precision values for the training and development data sets are reported when the reference concept is at the first rank after re-ranking the semantically most similar top (k = 5, 10, 15, 20, 25, 50) concepts

We also investigated the effects of using different similarity/distance metrics, word mover’s distance (WMD) and cosine similarity. The results show that the system with cosine similarity achieved better precision scores than the system with WMD on both the training (WMD: 58.6%; Cosine: 60.1%) and development (WMD: 49.0%; Cosine: 62.9%) data sets.

Table 7 shows the effect of the parameter *w*, which is used in Equation 1 to give weights to the most informative words (head of the noun phrase) with the ultimate aim to calculate the similarity between the named entity mention phrases and the reference ontology terms. As the results show, for *w*=0.25 our proposed approach obtains higher precision on both the training and the development sets.

**Table 7:** Results for the system with different weights for the most informative words (w = 0, 0.25, 0.50, 0.75)

w	Train	Dev
0	0.614	0.655
0.25	0.648	0.677
0.50	0.648	0.669
0.75	0.632	0.661

Precision values for the training and development data sets are reported

During the error analysis of the proposed system with syntactic re-ranking on the training and development sets, we realized the existence of falsely normalized mentions, which are possessive prepositional phrases (PPP). These phrases include compound noun phrases in the *“NP of NP”* form. For example, the entity mention *“throats of two healthy children”* is composed of two noun phrases *“throats”* and *“two healthy children”*, where the first NP *“throats”* is the only informative NP for normalizing the entity mention to the correct concept *“OBT:000374 throat”*. As a result of this fact, a syntax rule is added before re-ranking to strip the non-informative words following “of” from the entity mentions, if they are possessive prepositional phrases.

### Adverse drug reactions

Although experimental results showed that the new system with syntactic re-ranking obtained higher precision scores on both data sets than the system without syntactic re-ranking, the improvement of the new system on the Text Analysis Conference 2017 Adverse Drug Reaction (ADR) data set is lower compared to the improvement that is achieved on the BioNLP Shared Task 2016 Bacteria Biotopes data set. Error analysis revealed two main sources of errors, which are more prevalent in the ADR data set. The first source of errors is the usage of abbreviations and acronyms as entity mentions, which are hard to normalize without incorporating the context of the mentions. For example, in the training set, there are entity mentions such as *“sjs”* and *“ten”*, which are acronyms that should be normalized to the corresponding concepts *“Stevens-Johnson syndrome”* and *“Toxic epidermal necrolysis”* in the MedDRA dictionary. Rare words are the second source of errors. Although the word embedding model, which is used to calculate the semantic similarities, has been learned from PubMed articles, there may still exist out of vocabulary words, which are rare. For example, for the ADR mention *“Neoscytalidium infections”*, the *“Neoscytalidium”* word does not exist in the model that is used to calculate the word embeddings. In that case, the semantically most related concepts are found incorrectly by the proposed system considering only the existing word *“infections”* as *“Nosocomial infection”*, *“Opportunistic infection”* and *“Granulicatella infection”*, while the correct concept is *“Neoscytalidium infection”*.

## Conclusion

In this study, we introduce an unsupervised approach for biomedical entity normalization through an ontology by utilizing word embeddings and syntactic re-ranking. The proposed approach is applied to the normalization problem of the habitat entities through the Onto-Biotope ontology and the adverse drug reaction entities to the MedDRA dictionary, and tested on the BioNLP Shared Task 2016 Bacteria Biotopes data set and the Text Analysis Conference 2017 Adverse Drug Reaction data set, respectively. The new system with syntactic re-ranking obtained higher precision scores on both data sets than the system without syntactic re-ranking. Furthermore, the system achieved a precision score of 65.9% on the BioNLP Shared Task 2016 Bacteria Biotopes data set, which is 2.9 percentage points above the current state-of-the-art, demonstrating that it is as effective as supervised and semi-supervised approaches for biomedical named entity normalization.

Our proposed approach with syntactic re-ranking (named as the BOUNEL system) uses the Stanford Parser, which is a supervised parser. However, BOUNEL is unsupervised in the sense that it does not require training data manually annotated with entity mentions and their corresponding concepts in the ontology. Furthermore, the Stanford Parser has not been re-trained using biomedical data, but the off-the-shelf parser pre-trained with the Penn Treebank has been used. Therefore, the proposed approach can be easily adapted for normalizing different types of biomedical entities.

As future work, we will investigate incorporating the context of the reference entity mentions in text into the vector representations. Error analysis over the training sets revealed that the proposed approach is more successful for the normalization of entity mentions whose constituent words have semantic meanings, compared to the entity mentions which contain abbreviations, acronyms, or rare words. We believe that incorporating context information may improve the performance of the system for such entity mentions.

## Abbreviations

ADR: Adverse drug reactions
BB: Bacteria biotopes
BioNLP: Biomedical natural language processing
MedDRA: Medical dictionary for regulatory activities
NLP: Natural language processing
TAC: Text analytics conference
TF-IDF: Term frequency-inverse document frequency
